# Association between the docosahexaenoic acid to arachidonic acid ratio and acute coronary syndrome: a multicenter observational study

**DOI:** 10.1186/s12872-016-0299-y

**Published:** 2016-07-07

**Authors:** Yuji Nishizaki, Kazunori Shimada, Shigemasa Tani, Takayuki Ogawa, Jiro Ando, Masao Takahashi, Masato Yamamoto, Tomohiro Shinozaki, Tetsuro Miyazaki, Katsumi Miyauchi, Ken Nagao, Atsushi Hirayama, Michihiro Yoshimura, Issei Komuro, Ryozo Nagai, Hiroyuki Daida

**Affiliations:** Department of Cardiology, Juntendo University Graduate School of Medicine, 2-1-1 Hongo Bunkyo-ku, Tokyo, 113-8421 Japan; Department of Cardiology, Nihon University Hospital, 1-6 Kanda Surugadai Chiyoda-ku, Tokyo, 101-8309 Japan; Divison of Cardiology, Department of Internal Medicine, The Jikei University School of Medicine, 3-25-8, Nishi-Shimbashi Minato-ku, Tokyo, 105-8461 Japan; Department of Cardiovascular Medicine, Graduate School of Medicine, The University of Tokyo, 7-3-1 Hongo Bunkyo-ku, Tokyo, 113-8655 Japan; Department of Internal Medicine, Tokyo Takanawa Hospital, 3-10-11, Takanawa Minato-ku, Tokyo, 108-8606 Japan; Department of Biostatistics, School of Public Health, The University of Tokyo, 7-3-1 Hongo Bunkyo-ku, Tokyo, 113-0033 Japan; Division of Cardiology, Department of Medicine, Nihon University School of Medicine, 30-1 Ohyaguchi Kamichou Itabashi-ku, Tokyo, 173-8610 Japan; Jichi Medical University, 3311-1 Yakushiji, Shimotsuke-shi, Tochigi-ken 329-0498 Japan

**Keywords:** Acute coronary syndrome, Arachidonic acid, Docosahexaenoic acid, Eicosapentaenoic acid, DHA/AA ratio, EPA/AA ratio, Polyunsaturated fatty acids

## Abstract

**Background:**

A low eicosapentaenoic acid (EPA) to arachidonic acid (AA) ratio is a known risk for acute coronary syndrome (ACS). However, the association between the docosahexaenoic acid (DHA) to AA ratio and ACS remains unclear. This study aimed to assess the association between the DHA/AA ratio and ACS by patient characteristics.

**Methods:**

We enrolled 1733 patients and evaluated the serum levels of polyunsaturated fatty acids in 5 cardiology departments in a metropolitan area of Japan. We assessed the relationship between the DHA/AA ratio (median cut-off value: 0.903) and ACS according to the following 10 subgroups: sex, age, diabetes mellitus, hypertension, dyslipidemia, smoking history, family history of ischemic heart disease, chronic kidney disease, obesity, and history of coronary revascularization.

**Results:**

Interaction tests in the 10 subgroup analyses revealed a significant difference for adjusted log odds ratios between male and females (*p* = 0.01), and those with and without hypertension (*p* = 0.06). Especially in the subgroup based on sex difference, a high DHA/AA ratio was significantly associated with a low risk of ACS among men (adjusted odds ratio = 0.389; 95 % confidence interval: 0.211–0.716). In contrast, a reverse association was found among women, although this was not statistically significant (adjusted odds ratio = 3.820; 95 % confidence interval: 0.718–20.325).

**Conclusions:**

The association between the DHA/AA ratio and ACS differed by clinical characteristic. Notably, patients with a low DHA/AA ratio had a higher risk of ACS than those with a high DHA/AA ratio, and this was significant for men in particular.

## Background

Previous studies have demonstrated the beneficial effect of omega-3 polyunsaturated fatty acids (PUFAs) for preventing coronary events [1, 2]. In particular, several studies have reported that the eicosapentaenoic acid (EPA) to arachidonic acid (AA) ratio is a useful marker for estimating the risk of coronary events [3–5]. We recently reported that a low EPA/AA ratio was significantly associated with acute coronary syndrome (ACS) in a cohort of 1119 participants without any history of coronary revascularization or myocardial infarction [6]. However, the association between the docosahexaenoic acid (DHA) to AA ratio and coronary artery disease (CAD), particularly ACS, remains unclear. Although EPA and DHA are both omega-3 PUFAs, each may have different physiological and pharmacological effects [7]. We hypothesized that the effect of DHA/AA ratio for ACS could differ by patient characteristics, and that this difference might attenuate the association between the DHA/AA ratio and ACS when conducting analyses by ignoring the statistical interaction of the characteristics.

## Methods

### Study population

The present study was a multicenter observational cross-sectional study performed at four university hospitals and one community hospital located in Tokyo, Japan [6]. We enrolled 1733 patients who had their serum PUFA levels evaluated at these five centers from January 2004 to May 2011. The cohort included 151 patients with ACS and 1582 patients without ACS. Acute myocardial infarction was defined as an increase in the creatine kinase MB fraction or troponin T in patients with ischemic symptoms and/or typical electrocardiographic ST elevation. Unstable angina was defined as angina at rest or accelerated exertional angina combined with typical electrocardiographic ST-changes and an increased requirement for anti-ischemic therapy [8]. Patients were excluded if they were receiving hemodialysis or were currently taking EPA, and if they had concomitant congestive heart failure, severe liver dysfunction, or other systemic diseases, including malignancy and collagen disease. This study was approved by the Institutional Ethics Committees of each hospital, and all subjects gave informed consent.

### Measurements

We assessed the coronary risk factors and evaluated serum EPA, DHA, dihomo-gamma-linolenic acid (DGLA), and AA levels. We also measured the following laboratory parameters: total cholesterol, triglycerides (TG), low-density lipoprotein (LDL)-cholesterol, high-density lipoprotein (HDL)-cholesterol, hemoglobin A1c, serum creatinine (Cr), and estimated glomerular filtration rate (eGFR). The eGFR was calculated on the basis of the Japanese equation that utilizes serum Cr, age, and sex, as follows: eGFR (ml/min/1.73 m^2^) = 194 × Cr^−1.094^ × age^−0.287^ (female × 0.739) [9]. Serum EPA, DHA, DGLA, and AA levels were measured at an external laboratory (SRL, Inc., Tokyo, Japan). Blood samples were obtained from patients either during admission or at the laboratory of the outpatient clinic.

### Statistical analyses

We assessed the relationship between the DHA/AA ratio (at a median cut-off value of 0.903) and ACS in 10 different subgroups as follows: sex, age, diabetes mellitus (DM), hypertension (HT), dyslipidemia (DL), smoking history, family history of ischemic heart disease (IHD), chronic kidney disease (CKD), obesity defined as body mass index (BMI) ≥25, and history of coronary revascularization (percutaneous coronary intervention or coronary artery bypass grafting). To adjust for clinical characteristics other than the stratification variable for each subgroup, we fitted multivariable logistic regression models that included the following variables: age, BMI, serum Cr (continuous covariates), sex, smoking history, family history of IHD, HT, DM, or DL, a history of coronary revascularization (binary covariates), and the EPA/AA ratio. The EPA/AA ratio was divided into three tertiles for the present study, in line with our previous report that analyzed data extracted from the same study population [6]. Patients with missing data for any variable were excluded from the multivariable analysis. The alpha level for statistical tests was set at 0.05 and all confidence intervals were presented with their 95 % confidence levels.

The differences in associations (adjusted log odds ratios) between DHA/AA ratio and ACS from different subgroups were evaluated by the approximate formula for testing interaction [10]. Because of the limited power of the interaction test [11] and its nature as a preliminary test for statistical modeling [12], we considered a significance level of 0.10 to reflect a significant difference between subgroups. All analyses were performed with SAS version 9.2 (Cary, NC, USA).

## Results

Tables 1 shows the clinical characteristics and laboratory data for all patients by high and low DHA/AA ratios (≥0.903 and <0.903, respectively). Among the high DHA/AA ratio group (*n* = 867), the incidence of ACS was 7.50 % (65/867), compared with 9.93 % (86/866) in the low DHA/AA ratio group (*n* = 866). The crude odds ratio for ACS was 0.735 [95 % confidence interval (CI): 0.525, 1.030, *p* = 0.07] based on a DHA/AA ratio ≥0.903 versus <0.903. The adjusted odds ratio from the multivariable logistic model including all variables as regression covariates was 0.523 (95 % CI: 0.300, 0.910, *p* = 0.022).

**Table 1:** Baseline clinical characteristics and laboratory data

	All patients (*n* = 1733)	High DHA/AA ≥0.903 (*n* = 867)	Low DHA/AA <0.903 (*n* = 866)	*P*-value
Age (years)	64.5 ± 11.3	67.1 ± 9.2	61.8 ± 12.5	<0.01^a^
Male	77.8 %	79.4 %	76.2 %	0.11
Body mass index (kg/m^2^)	24.4 ± 3.5	24.4 ± 3.1	24.4 ± 3.8	0.97
Hypertension	73.1 %	75.7 %	70.6 %	0.01^a^
Diabetes mellitus	39.2 %	39.8 %	38.7 %	0.63
Dyslipidemia	70.4 %	70.6 %	70.2 %	0.86
Family history of ischemic heart disease	18.5 %	19.1 %	17.9 %	0.50
Smoking	45.1 %	47.6 %	42.5 %	0.03^a^
History of coronary revascularization (PCI or CABG)	27.7 %	26.8 %	28.6 %	0.38
Total cholesterol (mg/dl)	190.7 ± 35.8	190.7 ± 34.2	190.7 ± 37.4	0.98
Triglycerides (mg/dl)	146.8 ± 95.5	155.4 ± 100.2	138.4 ± 90.1	<0.01^a^
Low-density lipoprotein cholesterol (mg/dl)	111.2 ± 30.8	111.1 ± 29.9	111.2 ± 31.8	0.94
High-density lipoprotein cholesterol (mg/dl)	51.7 ± 16.5	50.0 ± 15.1	53.4 ± 17.6	<0.01^a^
Hemoglobin A1c (%)	6.3 ± 1.1	6.2 ± 0.9	6.3 ± 1.2	0.44
Serum creatinine (mg/dl)	0.88 ± 0.29	0.88 ± 0.27	0.87 ± 0.31	0.41
Estimated glomerular filtration rate (ml/min/1.73 m^2^)	68.7 ± 17.7	67.0 ± 16.4	70.2 ± 18.7	<0.01^a^
EPA (μg/dl)	73.9 ± 44.0	93.5 ± 47.9	54.2 ± 28.5	<0.01^a^
EPA/AA				<0.01^a^
Mean	0.49 ± 0.30	0.66 ± 0.32	0.32 ± 0.17	
Median	0.425	0.588	0.290	
DHA (μg/dl)	143.4 ± 52.8	170.9 ± 53.0	115.9 ± 35.3	<0.01^a^
DHA/AA				<0.01^a^
Mean	0.95 ± 0.38	1.22 ± 0.36	0.68 ± 0.15	
Median	0.903	1.143	0.706	
DGLA (μg/dl)	33.2 ± 12.3	30.8 ± 11.5	35.6 ± 12.6	<0.01^a^
AA (μg/dl)	157.4 ± 49.5	142.1 ± 33.8	172.8 ± 57.4	<0.01^a^
Statins	53.1 %	48.7 %	57.5 %	<0.01^a^
Antiplatelet agents	62.7 %	67.5 %	58.0 %	<0.01^a^
Renin angiotensin system inhibitor (ACE-I and ARB)	50.7 %	50.1 %	51.4 %	0.59
Calcium channel blockers	45.6 %	47.8 %	43.5 %	0.08
Beta blockers	38.6 %	39.6 %	37.6 %	0.43
Hypoglycemic agents	21.1 %	21.0 %	21.1 %	0.95

Values are mean ± standard deviation, or percentage, ^a^indicates statistical signficance, PCI = Percutaneous coronary intervention, *CABG* coronary artery bypass grafting, *EPA/AA* eicosapentaenoic acid to arachidonic acid ratio, *DHA/AA* docosahexaenoic acid to arachidonic acid ratio, *DGLA* dihomo-gamma-linolenic acid, *ACE-I* angiotensin converting enzyme inhibitor, *ARB* angiotensin II receptor blocker

In the high DHA/AA ratio group, the patient age, TG levels, and the prevalence of HT and smoking were significantly higher than in the low DHA/AA ratio group, whereas, the opposite was found in these groups regarding the HDL-cholesterol levels. In addition, the use of statins was significantly lower, and the use of antiplatelet agents was significantly higher in the high DHA/AA ratio group than in the low DHA/AA ratio group.

Serum AA level was significantly higher in patients with statin treatment than in patients without statin treatment (160.3 ± 40.9 vs. 154.1 ± 57.6 μg/dl, P = 0.01). Conversely, serum AA level was significantly lower in patients with antiplatelet agent treatment (152.5 ± 38.4 vs. 165.7 ± 63.2 μg/dl, *P* < 0.01), beta blocker treatment (152.9 ± 39.6 vs. 160.3 ± 54.7 μg/dl, *P* < 0.01), and hypoglycemic agent treatment (151.9 ± 40.1 vs. 158.9 ± 51.7 μg/dl, *P* = 0.01) than in patients without these treatments.

Table 2 shows the baseline clinical characteristics according to gender. The patient age, the levels of total cholesterol, LDL-cholesterol, and HDL-cholesterol were significantly higher in the female group than in the male group. The prevalence of DM, smoking, and family history of CAD as well as the use of antiplatelet agents and beta blockers was significantly lower in the female group than in the male group. The levels of BMI, TG, hemoglobin A1c, and Cr were significantly lower in the high DHA/AA ratio group than in the low DHA/AA ratio group.

**Table 2:** Baseline clinical characteristics according to gender

	Male (*n* = 1348)	Female (*n* = 385)	*P*-value
Age (years)	63.6 ± 11.1	67.5 ± 11.5	<0.01^a^
Body mass index (kg/m^2^)	24.5 ± 3.2	24.0 ± 4.3	0.02^a^
Hypertension	72.6 %	75.1 %	0.36
Diabetes mellitus	41.5 %	31.4 %	<0.01^a^
Dyslipidemia	70.5 %	70.1 %	0.89
Family history of ischemic heart disease	20.0 %	13.2 %	<0.01^a^
Smoking	53.6 %	47.6 %	<0.01^a^
History of coronary revascularization (PCI or CABG)	31.2 %	15.6 %	<0.01^a^
Total cholesterol (mg/dl)	187.1 ± 34.8	202.7 ± 36.8	<0.01^a^
Triglycerides (mg/dl)	151.8 ± 99.9	130.3 ± 77.6	<0.01^a^
Low-density lipoprotein cholesterol (mg/dl)	109.8 ± 30.3	115.6 ± 32.2	<0.01^a^
High-density lipoprotein cholesterol (mg/dl)	48.8 ± 14.3	61.3 ± 19.4	<0.01^a^
Hemoglobin A1c (%)	6.3 ± 1.1	6.1 ± 1.0	0.02^a^
Serum creatinine (mg/dl)	0.93 ± 0.29	0.71 ± 0.23	<0.01^a^
Estimated glomerular filtration rate (ml/min/1.73 m^2^)	68.8 ± 17.4	68.4 ± 18.7	0.72
EPA (μg/dl)	73.7 ± 43.2	74.4 ± 47.0	0.76
EPA/AA	0.50 ± 0.31	0.46 ± 0.29	0.01^a^
DHA (μg/dl)	141.8 ± 52.5	149.0 ± 53.2	0.01^a^
DHA/AA	0.96 ± 0.40	0.92 ± 0.33	0.06
DGLA (μg/dl)	32.7 ± 12.5	35.0 ± 11.5	<0.01^a^
AA (μg/dl)	154.4 ± 51.3	167.8 ± 41.3	<0.01^a^
Statins	53.0 %	53.2 %	0.95
Antiplatelet agents	67.9 %	44.7 %	<0.01^a^
Renin angiotensin system inhibitors (ACE-I and ARB)	50.7 %	50.6 %	1.00
Calcium channel blockers	44.4 %	50.1 %	0.04^a^
Beta blockers	41.7 %	27.8 %	<0.01^a^
Hypoglycemic agents	22.0 %	17.9 %	0.08

Values are mean ± standard deviation, or percentage, ^a^indicates significance, *PCI* Percutaneous coronary intervention, *CABG* coronary artery bypass grafting, *EPA/AA* eicosapentaenoic acid to arachidonic acid ratio, *DHA/AA* docosahexaenoic acid to arachidonic acid ratio, *DGLA* dihomo-gamma-linolenic acid, *ACE-I* angiotensin converting enzyme inhibitor, *ARB* angiotensin II receptor blocker

Table 3 shows the relationship between the DHA/AA ratio (≥0.903/<0.903) and ACS in the various subgroups. From interaction tests in 10 different subgroup analyses, the most significant difference in the adjusted log odds ratios was between men and women (*p* = 0.01), followed by the difference between those with and without HT (*p* = 0.06). A high DHA/AA ratio was significantly associated with a low risk of ACS among men in particular (adjusted odds ratio = 0.389; 95 % CI: 0.211–0.716), whereas it was associated with a high risk of ACS in women, although this latter result was not statistically significant (adjusted odds ratio = 3.820; 95 % CI: 0.718–20.325). These associations were in opposite directions, but of similar magnitude (i.e., 1/0.389 = 2.57). In all of the other groups, except for the female subgroup and the subgroup without DL, a high DHA/AA ratio (≥0.903) tended to have a negative association for ACS (Table 3).

**Table 3:** Relationship between DHA/AA (≥0.903) and ACS, subgroup analysis

Subgroup	DHA/AA	ACS	N	Prevalence	Crude OR (95 % CI)	Adjusted OR(95 % CI)	P-value for interaction
Male	≥0.903	47	688	6.8 %	0.572 (0.391, 0.838)	0.389 (0.211, 0.716)	0.01^a^
	<0.903	75	660	11.4 %			
Female	≥0.903	18	179	10.1 %	1.982 (0.910, 4.317)	3.820 (0.718, 20.325)	
	<0.903	11	206	5.3 %			
Age ≥65	≥0.903	43	539	8.0 %	0.842 (0.528, 1.343)	0.658 (0.329, 1.317)	0.23
	<0.903	35	375	9.3 %			
Age <65	≥0.903	22	328	6.7 %	0.620 (0.368, 1.044)	0.312 (0.115, 0.846)	
	<0.903	51	491	10.4 %			
Diabetes mellitus, yes	≥0.903	25	345	7.2 %	0.576 (0.341, 0.973)	0.424 (0.197, 0.915)	0.31
	<0.903	40	335	11.9 %			
Diabetes mellitus, no	≥0.903	40	522	7.7 %	0.875 (0.562, 1.361)	0.758 (0.338, 1.700)	
	<0.903	46	531	8.7 %			
Hypertension, yes	≥0.903	51	656	7.8 %	0.819 (0.552, 1.216)	0.706 (0.377, 1.320)	0.06^a^
	<0.903	57	611	9.3 %			
Hypertension, no	≥0.903	14	211	6.6 %	0.554 (0.285, 1.078)	0.162 (0.039, 0.670)	
	<0.903	29	255	11.4 %			
Dyslipidemia, yes	≥0.903	46	612	7.5 %	0.656 (0.443, 0.973)	0.447 (0.237, 0.845)	0.15
	<0.903	67	608	11.0 %			
Dyslipidemia, no	≥0.903	19	255	7.5 %	1.013 (0.523, 1.961)	1.139 (0.368, 3.527)	
	<0.903	19	258	7.4 %			
Smoking history, yes	≥0.903	40	413	6.8 %	0.652 (0.420, 1.010)	0.475 (0.218, 1.031)	0.68
	<0.903	52	368	14.1 %			
Smoking history, no	≥0.903	25	454	5.5 %	0.795 (0.467, 1.355)	0.598 (0.265, 1.350)	
	<0.903	34	498	6.8 %			
Family history of IHD, yes	≥0.903	9	166	5.4 %	0.298 (0.134, 0.661)	0.621 (0.174, 2.219)	0.76
	<0.903	25	155	16.1 %			
Family history of IHD, no	≥0.903	56	701	8.0 %	0.925 (0.633, 1.351)	0.499 (0.266, 0.939)	
	<0.903	61	711	8.6 %			
Chronic kidney disease, yes	≥0.903	18	236	7.6 %	0.607 (0.317, 1.161)	0.738 (0.277, 1.963)	0.40
	<0.903	23	192	12.0 %			
Chronic kidney disease, no	≥0.903	21	439	4.8 %	0.582 (0.337, 1.002)	0.439 (0.219, 0.880)	
	<0.903	40	503	8.0 %			
Obesity (BMI ≥25), yes	≥0.903	23	319	7.2 %	0.707 (0.402, 1.242)	0.909 (0.376, 2.196)	0.19
	<0.903	31	313	9.9 %			
Obesity (BMI <25), no	≥0.903	42	516	8.1 %	0.777 (0.508, 1.189)	0.425 (0.206, 0.877)	
	<0.903	53	518	10.2 %			
History of coronary revascularization, yes	≥0.903	32	232	13.8 %	0.742 (0.452, 1.217)	0.488 (0.243, 0.979)	0.84
	<0.903	44	248	17.7 %			
History of coronary revascularization, no	≥0.903	33	635	5.2 %	0.752 (0.470, 1.203)	0.554 (0.211, 1.454)	
	<0.903	42	618	6.8 %			

^a^indicates statistical signficance for interaction (*P* < 0.10), OR = odds ratio, CI = confidence interval, ACS = acute coronary syndrome, DHA/AA = docosahexaenoic acid to arachidonic acid ratio, IHD = ischemic heart disease, BMI = body mass index

**Fig. 1:**
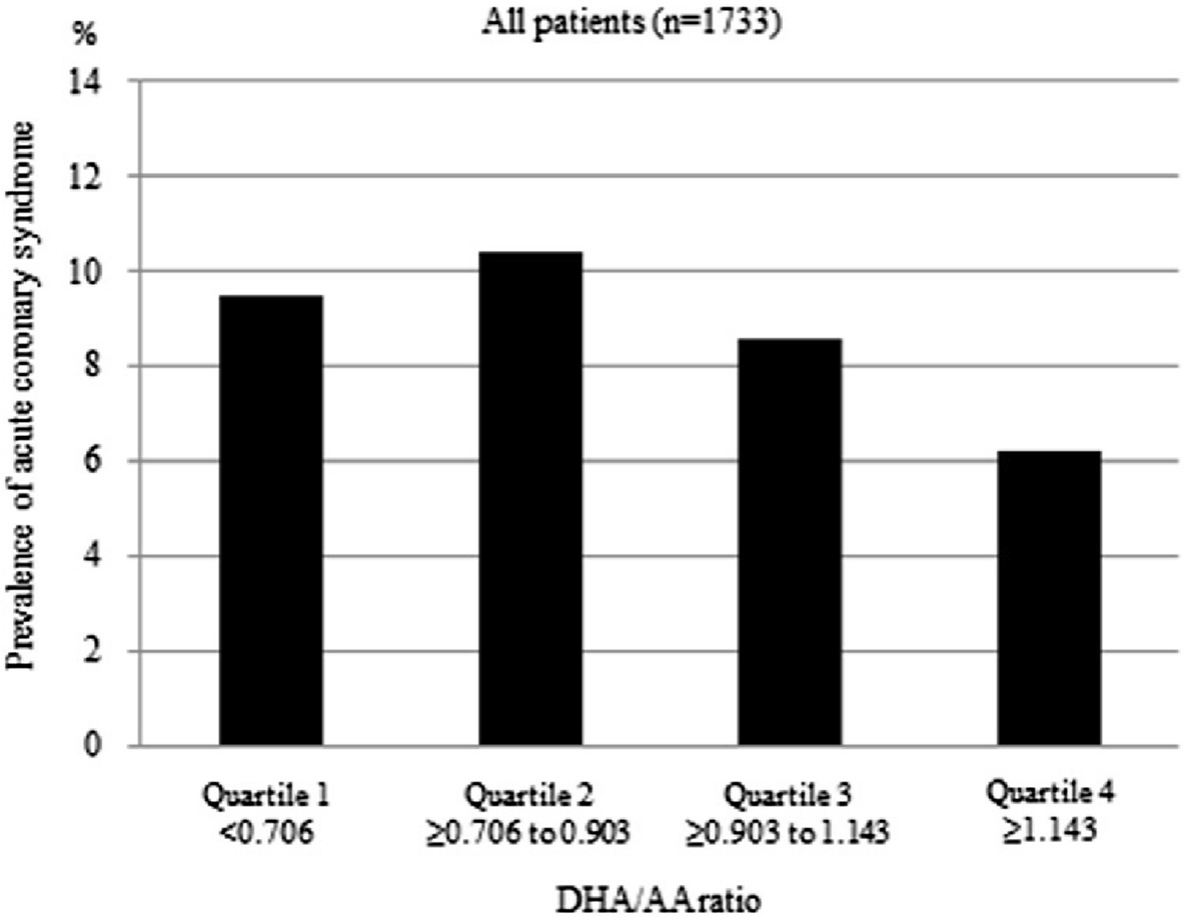
The prevalence of acute coronary syndrome by DHA to AA ratio quartile in all patients. DHA = docosahexaenoic acid, AA = arachidonic acid

**Fig. 2:**
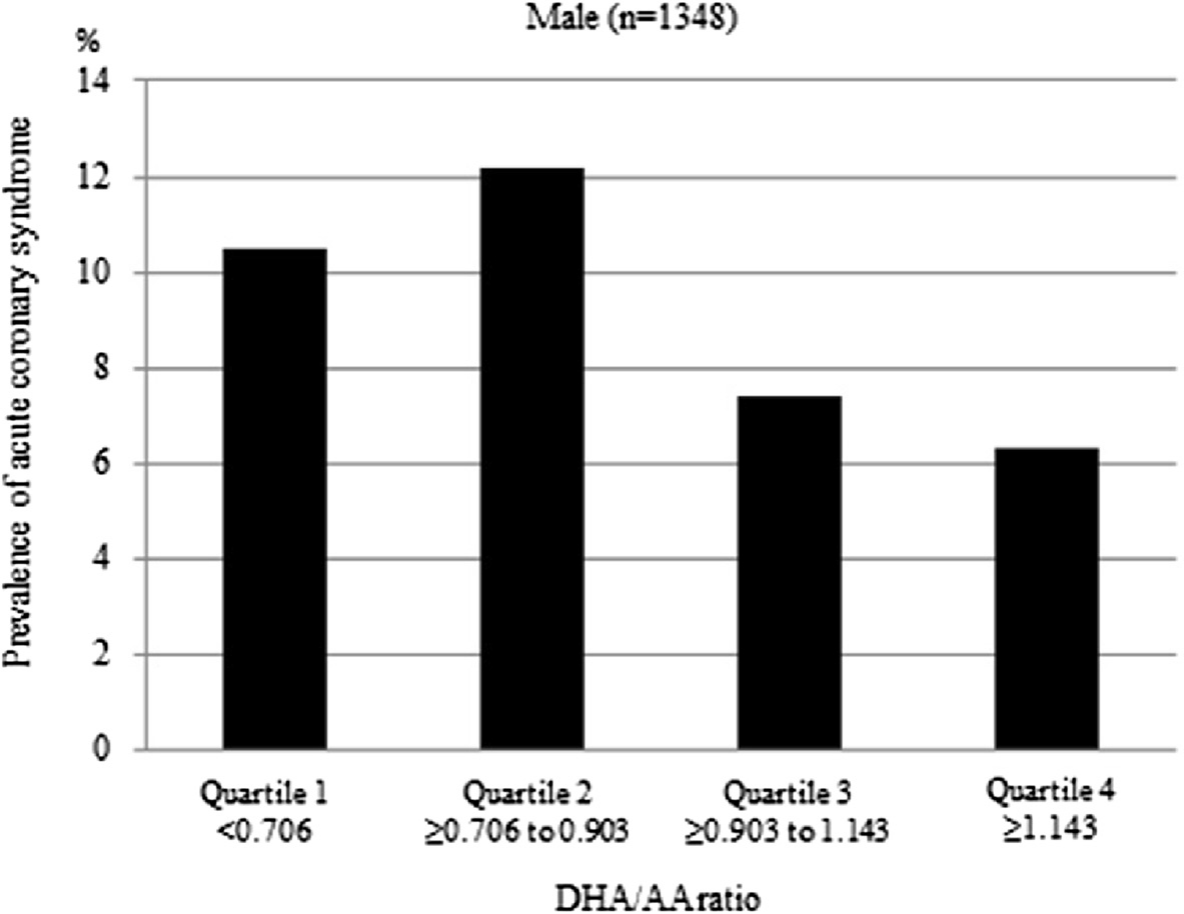
The prevalence of acute coronary syndrome DHA to AA ratio quartile in men. DHA = docosahexaenoic acid, AA = arachidonic acid

**Fig. 3:**
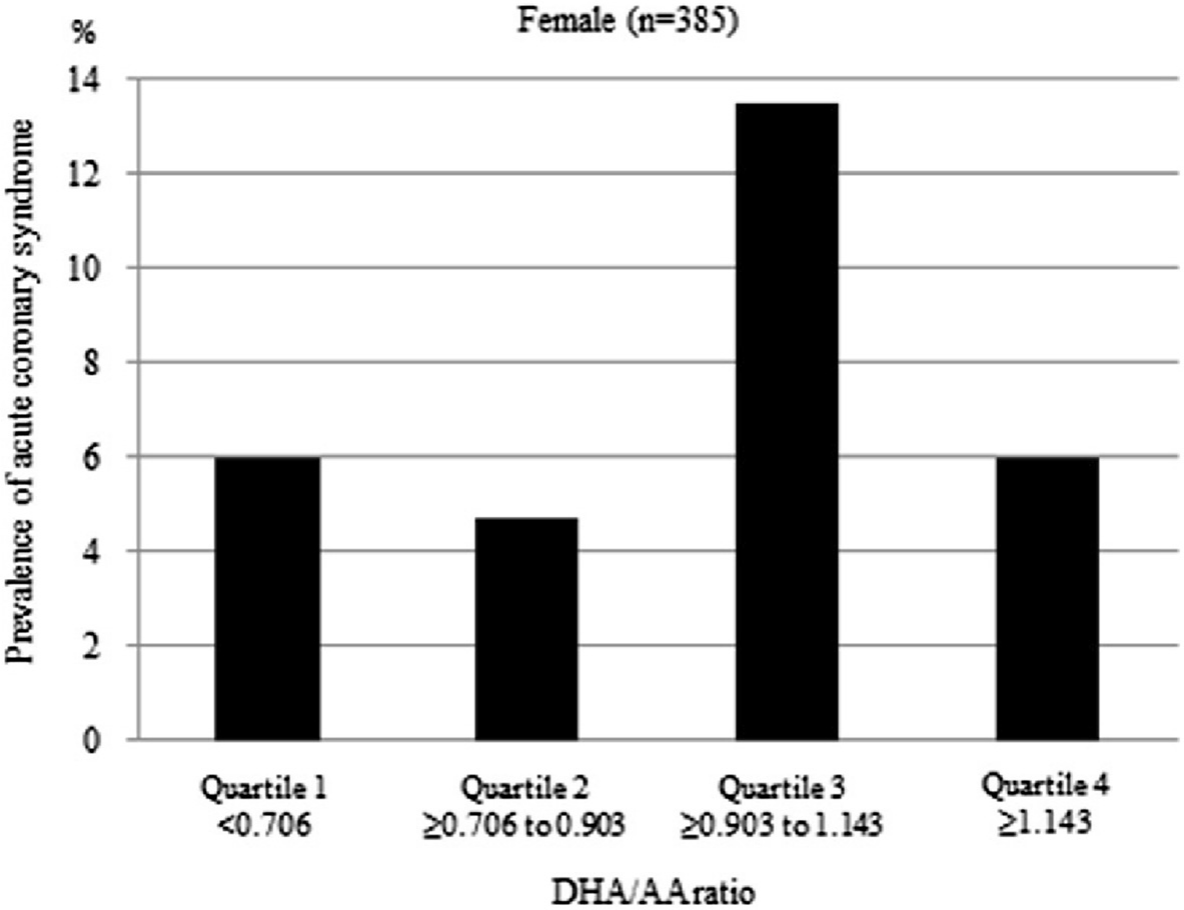
The prevalence of acute coronary syndrome by DHA to AA ratio quartile in women. DHA = docosahexaenoic acid, AA = arachidonic acid

Figure 1 shows the prevalence of ACS by a quartile of DHA/AA ratio in all patients. The prevalence of ACS in quartile 1 was 9.5 % (41/433), in quartile 2 was 10.4 % (45/433), in quartile 3 was 8.6 % (38/434), and in quartile 4 was 6.3 % (27/433). Figure 2 shows the prevalence of ACS by a quartile of DHA/AA ratio for men, revealing a prevalence of ACS in quartile 1 of 10.5 % (35/333), in quartile 2 of 12.2 % (40/327), in quartile 3 of 7.4 % (25/338), and in quartile 4 of 6.3 % (22/350). Figure 3 then shows the prevalence of ACS by a quartile of DHA to AA ratio for women, revealing a prevalence of ACS in quartile 1 of 6.0 % (6/100), in quartile 2 of 4.7 % (5/106), in quartile 3 of 13.5 % (13/96), and in quartile 4 of 6.0 % (5/83). Thus, there were clearly different trends of ACS risk for the DHA/AA ratio between men and women.

## Discussion

Previous studies have demonstrated that treatment with EPA can reduce the risk of CAD [1, 2]. The Japan EPA Lipid Intervention Study (JELIS) proved that EPA administration could reduce residual risk in statin-treated patients [1]. In a sub-analysis of the JELIS, EPA administration had a beneficial effect for coronary or cerebrovascular events, particularly in statin-treated patients with abnormal TG and HDL- cholesterol levels, as well as in patients with glucose intolerance, a history of stroke, and those with CAD [13–16]. These data suggested the importance of supplemental EPA for reducing any residual risk after statin treatment. However, the clinical usefulness of DHA treatment remains unclear, partially because no purified DHA-only agent has been launched in the clinical setting.

We report that a low EPA/AA ratio is associated with CAD and ACS, which is consistent with previous reports [3–6]. Therefore, an imbalance in the ratio of omega-3 to omega-6 PUFAs in the serum might be one of the residual risks. Several studies have reported the association between coronary events and the EPA/AA and DHA/AA ratios. Ninomiya et al*.* showed that a low EPA/AA ratio but not the DHA/AA ratio was associated with a risk of cardiovascular disease in subjects with higher levels of high-sensitivity C-reactive protein [4]. Domei et al. evaluated the relationship between the EPA/AA or DHA/AA ratios and major adverse cardiac events in patients who underwent elective percutaneous coronary intervention. They concluded that those with a higher EPA/AA ratio (>0.4037) had significantly lower major adverse cardiac events than those with a low EPA/AA ratio, but not DHA/AA, ratio [5]. Lee et al. demonstrated that high EPA levels rather than DHA were significantly associated with cardiovascular mortality [17]. EPA biologically competes with AA and works as a central substance for anti-inflammation reducing prostaglandins or leukotrienes, which are induced by AA. We believe that the clinical assessment of DHA/AA is also important because DHA and EPA have anti-inflammatory effects.

In contrast, a previous study has reported that high plasma DHA is associated with reduced progression of coronary atherosclerosis in patients with CAD [18]. In a clinical study using virtual histology intravascular ultrasound, the DHA/AA ratio had a stronger negative relationships with changes in plaque volume than the EPA/AA ratio in statin-treated patients with CAD [19]. Previous studies have also reported that the DHA level has a strong inverse association with intima-media thickness when compared with the EPA level [20, 21]. DHA rather than EPA has also been shown to lower ambulatory blood pressure and heart rate [22]. Moreover, other studies have demonstrated numerous beneficial effects of DHA, including microcirculation [23], antiplatelet [24], and anti-inflammation effects [25]. However, differences in the current results for EPA and DHA may be due to differences in participants, ethnic groups, and concomitant medications. As demonstrated in the present study, we may need to focus on the levels of both the EPA/AA ratio and the DHA/AA ratio.

Although EPA and DHA are both omega-3 PUFAs, more attention has been focused on the differences in their physiological and pharmacological effects [7]. Although the detailed mechanisms are yet to be clarified, both EPA and DHA have anti-inflammatory, anti-thrombotic, TG-lowering, inhibition of platelet aggregation, improvement of endothelial function, and plaque stabilization effects [26–29]. These beneficial effects might be additive for anti-atherogenesis in patients with high EPA/AA and DHA/AA ratios. Further investigation is needed to clarify these issues.

Several clinical studies have previously reported gender differences in the relationship between omega-3 PUFAs and cardiovascular mortality [17, 30, 31]. In 2013, The Risk and Prevention Study Collaboration Group conducted a double-blind, placebo-controlled clinical trial to evaluate the preventive effects of omega-3 PUFAs (EPA and DHA) on cardiovascular mortality in 12,513 patients with multiple cardiovascular risk factors [30]. Although null results were observed for the primary and secondary endpoints, a gender difference was observed in a subgroup analysis. Omega-3 PUFAs showed preventive effects for cardiovascular events in women (hazard ratio, 0.82; 95 % CI, 0.67–0.99) but not in men (hazard ratio, 1.04; 95 % CI, 0.92–1.17). Lee et al. reported a stronger association between high plasma EPA level and mortality reduction among women with acute myocardial infarction than among men [17]. In a Finnish study, higher fish consumption was related to a decreased risk of coronary heart disease among women, but no significant relationship was observed among men [31]. Although previous studies have reported that omega-3 PUFAs were significantly associated with cardiovascular events and mortality in women, we found a significant relationship between the DHA/AA ratio and ACS events in men. While gender differences can partially be explained by the effects of sex hormones, the precise mechanisms that lead to gender differences are not fully understood. It is also not clear whether gender altered the effects of omega-3 PUFAs on cardiovascular mortality. In particular, reports that focus on gender differences in the relationship between the DHA/AA ratio and cardiovascular events are rare.

We also assessed the association between serum AA level and medications and found that the administration of statins, antiplatelet agents, beta blockers, and hypoglycemic agents was significantly associated with serum AA levels. The relationship between statin administration and AA cascade has already been reported [32], and it is well known that aspirin prevents antithrombotic events through affecting the AA cascade; [33] thus, it could reduce serum AA levels. The relationships between beta blocker, hypoglycemic agents, and AA have still not been clarified and need further studies.

It has been reported that human immunodeficiency virus (HIV) infection is a prognostic indicator for recurrent thrombotic events [34]. However, we believe that it has a relatively low impact on the results of our study because the incidence of HIV infection is quite low in Japan.

There are several limitations of the present study. First, because it was conducted in the metropolitan Tokyo area, the results are not necessarily applicable to patients living in rural areas or foreign countries. Second, we were unable to prove a cause-effect relationship because of the cross-sectional design of our study. Third, as the duration of the study was over 7 years, medical advances during this period might have affected the study results. Fourth, assessment of dietary habits is important because plasma fatty acid profile depends upon their dietary habits. The assessment was not conducted in this study. However, PUFAs, such as EPA and DHA, are not sufficiently produced in the human body. We believe that the levels of essential plasma fatty acids, such as EPA and DHA, must be influenced by dietary habits. Fifth, fatty acid profiles were assessed only in four subsets (EPA, DHA, DGLA, and AA), and complete fatty acid profiles were not presented. Sixth, we have not conducted an assessment of inflammatory cytokines in this study. Inflammatory cytokines reduction in the plasma was observed. Dietary intake of omega-3 fatty acids correlates with the decrease in inflammatory cytokines in the plasma [35]. Seventh, the red blood cell lipid profile is known to have a significant correlation between serum lipid profile and ACS [36]. We should have evaluated between them. Eighth, because the data in the present study is male dominant (close to 80 %), a bias from the patient cohort or study design might be associated with these results. Ninth, in the present study, fasting blood samples from patients with ACS were collected during admission. In the early stage of ACS, the serum fatty acid profiles would possibly be altered [37]. Finally, we also enrolled the patients who had any coronary intervention and/or coronary artery bypass grafting due to stable angina. These patients may have less incidence of ACS because of their medical therapy. A large-scale multicenter prospective study is necessary to validate the results of this study.

The present study showed that the DHA/AA ratio was more effective for risk stratification due to the ACS events and that the relationship of EPA/AA ratio with ACS events was not significant. In our previous report [6], patients with a medical history of percutaneous coronary intervention, coronary artery bypass grafting, and myocardial infarction were excluded, whereas those with past coronary revascularization were included in the present study. In a meta-analysis of randomized placebo-controlled trials of EPA vs DHA intervention, both EPA and DHA reduced TG, with a greater reduction seen with DHA. DHA, but not EPA, also raised HDL-cholesterol [38]. There are several reported mechanisms by which the differences between EPA and DHA treatment may affect lipid metabolism. EPA induces the reduction of hepatic lipogenesis with a more potent peroxisome proliferator-activated receptor-alpha effect, whereas DHA enhances very-low-density lipoprotein (VLDL) lipolysis and increases HDL and large LDL particles [39]. Several studies demonstrate that DHA, but not EPA, reduces the apolipoprotein C-III concentration [40, 41]. Apolipoprotein C-III particularly inhibits the actions of lipoprotein lipase and hepatic lipase, which slow TG hydrolysis [42]. By regulating different hepatic transcription factors than EPA, DHA reduces apolipoprotein C-III production, resulting in enhanced conversion of VLDL to LDL to reduce TG [39, 43]. However, until now, there was no study that investigated the difference between EPA alone and EPA/DHA combination on clinical outcomes, including cardiovascular events and total mortality. The bio-reactive function of EPA or DHA may be different among the characteristically different groups of patients. In addition, the data of EPA/AA and DHA/AA ratio was not strongly, but moderately correlated (data not shown) in the present study. EPA and DHA may show a parallel function in the body, but data collected from a real clinical setting, as the present study, do not necessarily show a notable correlation. Further studies should be conducted to clarify these mechanisms. In the near future, we believe that EPA or DHA will be appropriately administered to patients, depending on their clinical background.

## Conclusions

The present study showed that the relationship between the DHA/AA ratio and ACS differed with the sex of participants who evaluated the serum levels of PUFAs in cardiology departments. Those men with low DHA/AA ratios had 2.57 times more ACS than those with a high DHA/AA ratio. In contrast, a reverse association of similar magnitude was found among women, although this was not statistically significant. In the present study, healthy subjects were not the focus of attention. Further studies are needed to clarify the association between DHA/AA ratio and the risk of ACS in apparently healthy subjects.

## Abbreviations

AA, arachidonic acid; ACS, acute coronary syndrome; BMI, body mass index; CAD, coronary artery disease; CI, confidence interval; CKD, chronic kidney disease; Cr, creatinine; DGLA, dihomo-gamma-linolenic acid; DHA, docosahexaenoic acid; DL, dyslipidemia; DM, diabetes mellitus; eGFR, estimated glomerular filtration rate; EPA, eicosapentaenoic acid; HDL, high-density lipoprotein; HIV, human immunodeficiency virus; HT, hypertension; IHD, ischemic heart disease; JELIS, Japan EPA Lipid Intervention Study; LDL, low-density lipoprotein; PUFAs, polyunsaturated fatty acids; TG, triglycerides; VLDL, very-low-density lipoprotein
